# Intramural Longitudinal Sigmoid Fistula

**DOI:** 10.5334/jbsr.3593

**Published:** 2024-06-25

**Authors:** Brecht Van Berkel, Vincent Sneyers, Geert Verswijvel

**Affiliations:** 1 UZ Leuven, Leuven, Belgium; 2 UZ Leuven, Leuven, Belgium; 3 ZOL Genk, Genk, Belgium

**Keywords:** Diverticulitis, CT, fistula

## Abstract

*Teaching point:* A longitudinal intramural fistula is a rare complication of diverticulitis that can be visualised by computed tomography (CT).

## Case History

**Figure 1:**
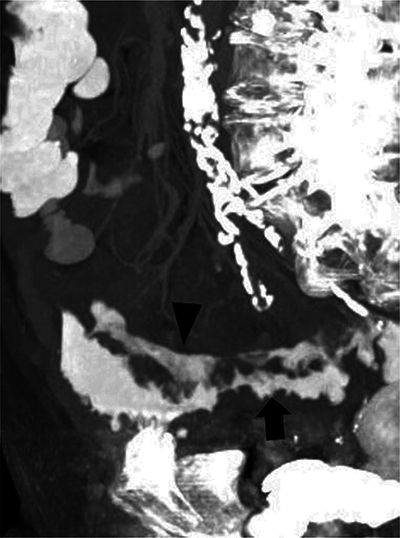
Maximum intensity projection imaging of the abdominal CT: two contrast trajectories are distinguishable, the intramural longitudinal fistula (arrowhead) and the normal intraluminal contrast of the sigmoid (arrow).

**Figure 2:**
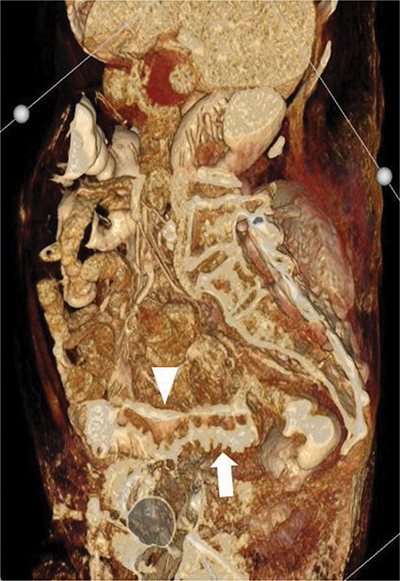
Volume-rendered three-dimensional reconstruction of the abdominal CT. The intramural longitudinal fistula is shown (arrowhead) to run parallel with the intraluminal contrast of the sigmoid (arrow).

A 90-year-old female patient with a medical history of diabetes mellitus type 2, hypothyroidism and total hip joint replacement presented with nausea, diarrhoea and left lower quadrant pain. Computed tomography (CT) demonstrated severe acute diverticulitis complicated with a longitudinal intramural fistula along the course of the proximal and distal sigmoid (Figures 1 and 2). Unfortunately, the patient developed acute renal failure and peritonitis and passed away 1 month later.

## Comments

Acute diverticulitis is one of the most frequent abdominal emergencies. The pathophysiology of diverticulitis is obstruction of the ostium of a diverticulum and subsequent inflammation. It is estimated that up to 25% of patients with diverticulosis will present with diverticulitis, which is uncomplicated in the majority of cases. CT is the imaging modality of choice for diagnosis and for the detection of complications, such as fistulas. Complications are perforation, abscess formation, bowel obstruction, pylephlebitis, haemorrhage and fistula formation. The latter occurs in approximately 14% of patients with complicated diverticulitis. Common types of fistulas related to diverticulitis include colovesical, coloenteric, colouterine and colovaginal fistulas. These fistulas are treated with surgery. An extremely rare type of fistula is the intramural longitudinal fistula, with exit and re-entrance ostium on the course of the same bowel segment. This type of fistula is rare and is only reported in a few cases [1].
